# Does long-term efficacy differ between infliximab and adalimumab after 1 year of continuous administration? A STROBE-compliant retrospective cohort study

**DOI:** 10.1097/MD.0000000000006635

**Published:** 2017-04-21

**Authors:** Haruka Otake, Satohiro Matsumoto, Hirosato Mashima

**Affiliations:** Department of Gastroenterology, Saitama Medical Center, Jichi Medical University, Saitama, Saitama Prefecture, Japan.

**Keywords:** adalimumab, Crohn disease, infliximab, loss of response

## Abstract

Although biologics are important inflammatory bowel disease therapies, loss of response (LOR) remains problematic. We evaluated LOR to biologics in our Crohn disease (CD) patients receiving biologics. Of 137 biologic-treated CD patients, 68 continuously receiving the same biologic type for at least 1 year were divided into 2 groups: infliximab (IFX) (n = 39) and adalimumab (ADA) (n = 29). Clinical courses were compared at biologic introduction and at 1 year. Both groups were retrospectively analyzed for LOR at and beyond 1 year after biologic introduction (study endpoint). Patients were then divided into LOR and non-LOR groups to identify factors predicting LOR. At 1 year after biologic introduction, decreases in CD activity index were 94 ± 105 in the IFX and 102 ± 89 in the ADA group, not significantly different. Blood test data did not differ between these groups. LOR occurred in 14 IFX and 5 ADA group patients. Event-free rates at 5 years after biologic introduction were 62% in the IFX and 61% in the ADA group. Patients achieving clinical remission 1 year after biologic introduction accounted for 69% of the IFX and 90% of the ADA group, while respective rates of secondary LOR at 5 years were 32% and 26%. C-reactive protein (CRP) at biologic introduction (odds ratio [OR], 1.5; 95% confidence interval [CI], 1.04–2.06; *P* = .02) and age at CD onset (OR, 1.1; 95% CI, 1.01–1.20; *P* = .03) predicted LOR. As to IFX and ADA efficacies after 1 year of administration, there were no significant differences in event-free rates for the 5 years after biologic introduction or the secondary LOR rate. CRP at biologic introduction and age at CD onset predicted LOR.

## 1 Introduction

In Japan, infliximab (IFX) was approved as a therapeutic agent for Crohn disease (CD) in 2002. Adalimumab (ADA), the second biologic agent, was approved for the treatment of CD in 2010. These biologics are not only highly effective for inducing and maintaining remission but also have a mucosal healing effect. IFX exerted a long-term effect on remission maintenance, that is, 54 weeks, in the ACCENT 1 (A Crohn Disease Clinical Trial Evaluating Infliximab in a New Long-term Treatment Regimen) trial.^[1]^ Likewise, ADA also produced a long-term effect on remission maintenance, again 54 weeks, in the CHARM (Crohn Trial of the Fully Human Antibody ADA for Remission Maintenance) trial.^[2]^

Although biologics are important therapeutic agents for inflammatory bowel diseases, the incidence of loss of response (LOR) remains a problem. In the ACCENT 1 trial, LOR to IFX was observed in approximately 30% of patients by week 54.^[1]^ The response to IFX was reportedly lost in 37% of the total 2236 CD patients receiving long-term administration of this biologic agent, and the annual rate of LOR was 13% per patient-year.^[3]^

Although 1 study found no differences between IFX and ADA in the proportions of patients who underwent surgical treatment and/or were hospitalized during 26 weeks of biological therapy,^[4]^ few studies have directly compared IFX and ADA in terms of long-term therapeutic effects and LOR. In the present study, we compared IFX and ADA to evaluate both their long-term efficacies and LOR to these agents in CD patients.

## 2 Materials and methods

### 2.1 Patients

**Figure 1:**
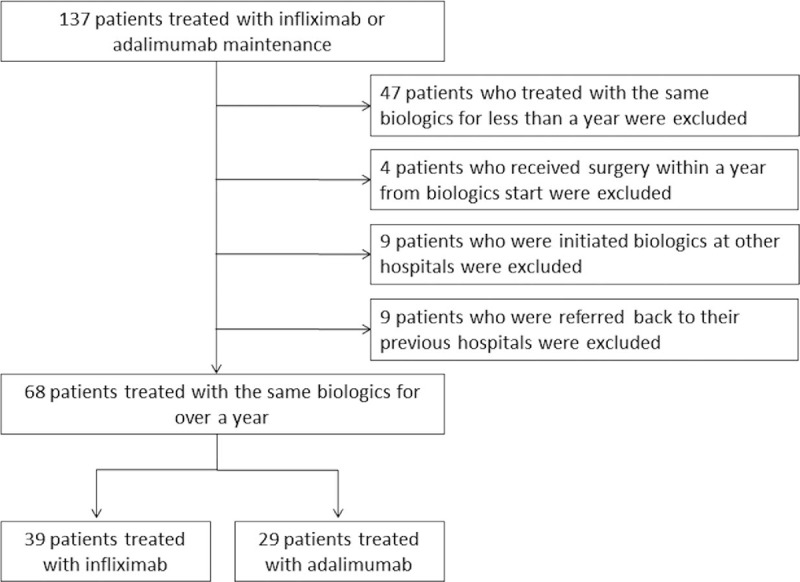
Flowchart of patient inclusion and exclusion.

Based on medical records, we enrolled 137 CD patients who had started biologic therapy and regularly visited Saitama Medical Center for follow-up examinations between 2004 and 2016. The inclusion criteria were at least 16 years of age and continuous administration of the same type of biologic agent for at least 1 year. The exclusion criteria were discontinuation of the biologic agent within 1 year after introduction, switching to another type of biologic within 1 year after introduction, biologic dose doubled within 1 year after introduction, transfer to another hospital within 1 year after introduction, and receive surgery within 1 year after induction. Ultimately, 68 patients (47 men and 21 women with a mean age of 24 ± 9 years at CD onset and a mean disease duration of 13.9 ± 8.8 years) were included in the analyses (Fig. 1).

IFX was administered at a dose of 5 mg/kg at weeks 0, 2, and 6, followed by a maintenance dose every 8 weeks. ADA was administered at doses of 160 mg at week 0 and 80 mg at week 2, followed by a maintenance dose of 40 mg every 2 weeks.

### 2.2 Comparison of efficacy for CD between infliximab and adalimumab

Treatment regimens and clinical courses were assessed in the 2 groups, those treated with IFX and ADA, at the time of introducing biologics and 1 year after introduction. Clinical symptoms were assigned scores based on the CD activity index (CDAI). Clinical remission was defined as CDAI < 150, LOR as CDAI ≥ 150, and C-reactive protein (CRP) >5 mg/L. Furthermore, the endpoint was the incidence of events caused by LOR in over 1 year after the introduction of biologics. The patients were followed up for maximum 6 years until March 2017. These 2 groups were retrospectively analyzed. Secondary LOR was defined as patients who achieved clinical remission at 1 year after the introduction of biologics and subsequently had LOR. The events attributed to LOR were an increase in the dose of biologics, switching to another type of biologic agent, additional treatment with prednisolone, hospitalization due to deterioration of CD status, and surgical treatment.

### 2.3 Comparison between loss of response and nonloss of response to biological therapy

The patients were divided into those with and without LOR to biologic agents (LOR and non-LOR groups). Background factors, treatment regimens, and clinical courses were then compared and analyzed. Furthermore, multivariate analysis was performed to identify predictive factors for LOR.

### 2.4 Ethical considerations

This study was approved by The Etiological Study Ethical Review Board of Saitama Medical Center, Jichi Medical University. As we produced and used only anonymized data, informed consent from the study subjects was not needed in any case.

### 2.5 Statistical analysis

Data are expressed as means ± standard deviation or percentage. The demographic characteristics of the study subjects were compared using the Student *t* test and Fisher exact test. The cumulative event-free rate was evaluated by the Kaplan–Meier method, and comparisons were made using the log-rank test. Factors identified as significant by univariate analysis (*P* < .1) were entered into a multivariate logistic regression analysis model. All data analyses were performed with StatView software (version 5.0; SAS Institute Inc., Cary, NC). Differences at *P* values of less than .05 were regarded as significant.

## 3 Results

In the 68 patients, the age distribution at CD onset peaked at 15 to 20 years, showing a trend similar to the results of other surveys in Japan. The observed disease types were the small intestine type in 19.1% of patients, the combined small and large intestine type in 66.2%, and the large intestine type in 14.7%. Intestinal fistula and anal fistula were observed in 23.5% and 26.5%, respectively. Bowel surgery had been performed in 33.8%, and surgery for anal fistula in 25.0%. Current smokers accounted for 29.4%. The concomitant treatments were mesalazine in 97.1%, immunomodulators in 52.9%, and elemental diet in 72.1%. Regarding background factors at the time of introducing biologics, there were no differences in age, sex, or disease duration between the IFX and ADA groups. No significant difference was observed between the 2 groups in the concomitant use of corticosteroids and immunomodulators (Table 1).

**Table 1:**
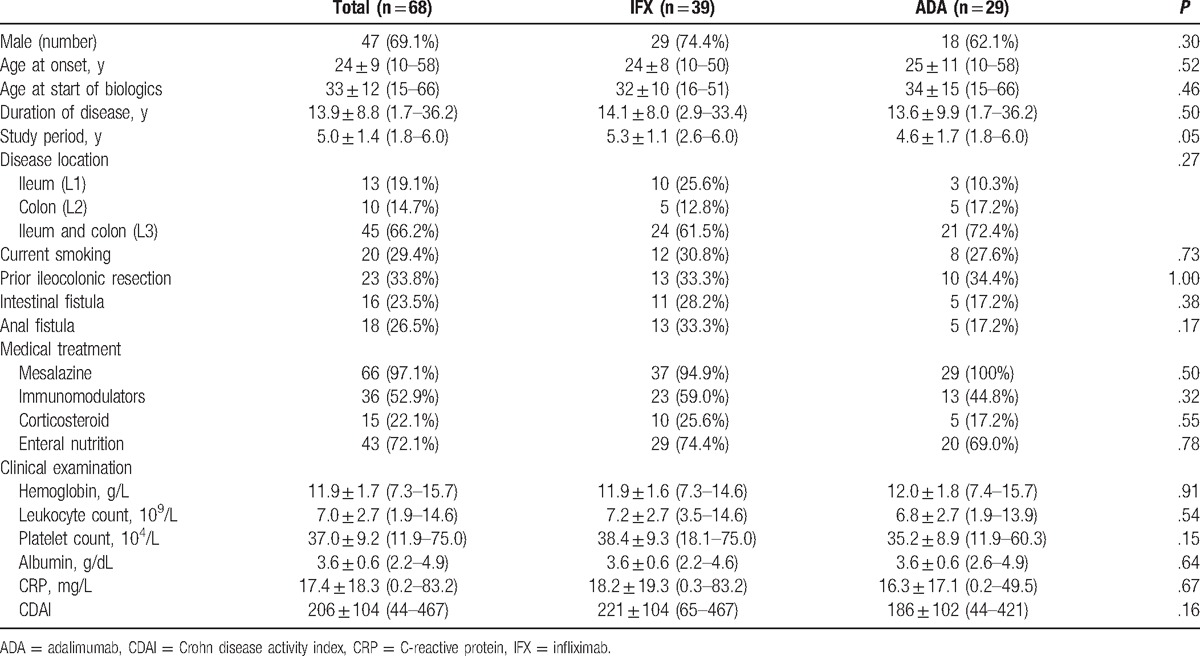
Baseline characteristics.

Regarding clinical effects at 1 year, CDAI was 221 ± 104 at the time of introducing biologics and 123 ± 68 at 1 year in the IFX group (*P* < .0001), while the corresponding values in the ADA group were 186 ± 102 and 84 ± 70 (*P* < .0001). Although CDAI decreased significantly in both groups, there was no significant difference between the 2 groups. Patients with LOR accounted for 35.9% (14/39) of the IFX and 17.2% (5/29) of the ADA group, showing no significant difference. Patients with new development of intestinal stenosis or worsening of preexisting intestinal stenosis accounted for 12.8% of the IFX and 10.3% of the ADA group, showing no significant difference. The numbers of patients who achieved clinical remission (CDAI < 150) at 1 year were 27 (69.2%) and 26 (89.7%) in the IFX and ADA groups, respectively, showing no significant difference. Of these patients, 33.3% (9/27) in the IFX group and 19.2% (5/26) in the ADA group experienced secondary LOR (Table 2). The only observed adverse reactions were in the IFX group (2 patients; 2.9%). One patient each had arthralgia and peripheral neuropathy, neither of which was serious.

**Table 2:**
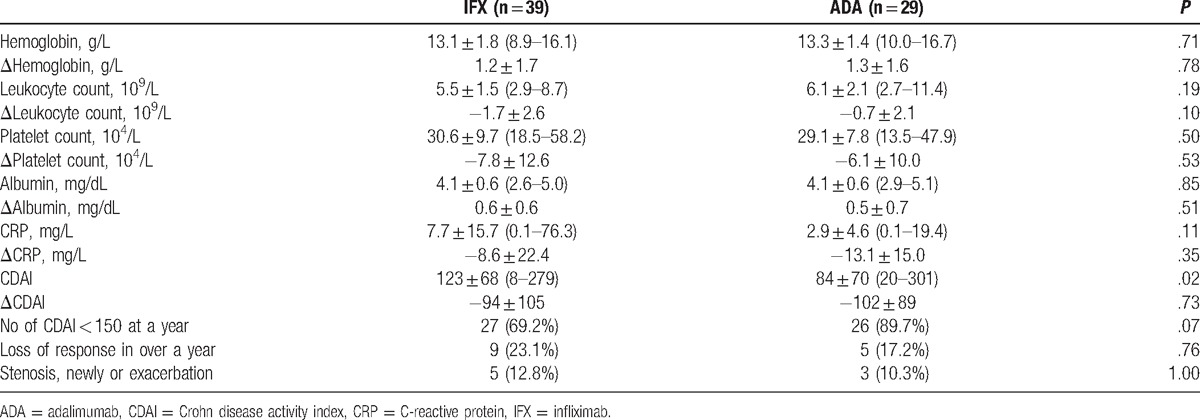
Outcomes at a year: comparison between the infliximab and the adalimumab groups.

**Figure 2:**
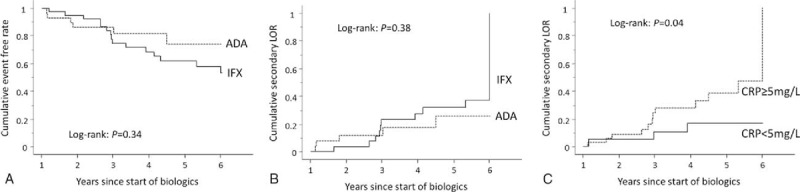
(A) Kaplan–Meier analysis of the cumulative event-free rate in the infliximab (IFX) and the adalimumab (ADA) groups. (B) Kaplan–Meier analysis of the cumulative secondary loss of response (LOR) rate in the IFX and the ADA groups. (C) Kaplan–Meier analysis of the cumulative secondary LOR rate according to C-reactive protein level at week 0.

The cumulative event-free rates were determined at 1 year, and later, after the introduction of biologic agents. The rates were 94.9%, 74.5%, and 61.6% at 2, 3, and 5 years, respectively, after introduction of IFX, while the corresponding rates in the ADA group were 85.9%, 81.4%, and 74.0% (Fig. 2A). The cumulative secondary LOR rates were 3.6%, 23.3%, and 31.8% at 2, 3, and 5 years after the introduction of IFX, while the corresponding rates in the ADA group were 11.7%, 17.2%, and 25.5%. No significant differences were detected in these rates (Fig. 2B). Then, because Hibi et al^[4]^ reported that a CRP level of more than 5 mg/L can serve as a predictive factor for subsequent LOR to IFX, the cumulative secondary LOR rates were analyzed employing a CRP cutoff level of 5 mg/L. The rates in patients with a CRP level of 5 mg/L or more were 8.9%, 28.2%, and 38.1% at 2, 3, and 5 years, whereas the rates in patients with a CRP level of less than 5 mg/L were 5.0%, 10.6%, and 17.0%, respectively. The differences were statistically significant (*P* = .04) (Fig. 2C).

**Figure 3:**
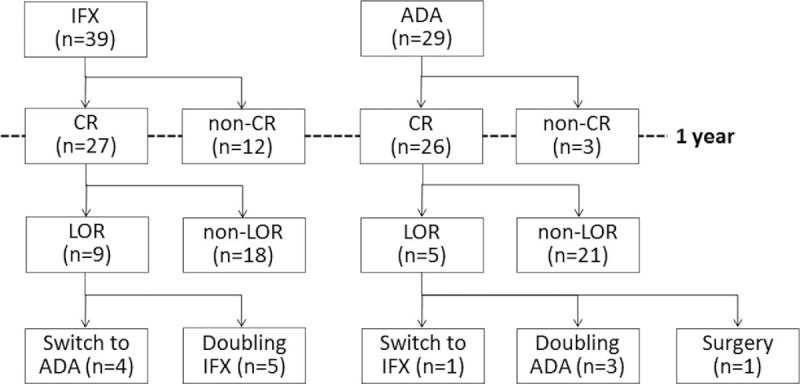
Flowchart of outcomes of Crohn disease patients treated with infliximab or adalimumab.

Regarding the clinical courses of patients with LOR, in the IFX group, the dose of IFX was increased to 10 mg/kg in 5 patients, and IFX was switched to ADA in 5 others. In the ADA group, in 1 patient each ADA was switched to IFX, intestinal resection was performed, and ADA administration was continued without changes (Fig. 3).

The subanalysis comparing the LOR group of 19 patients and the non-LOR group of 49 revealed no difference in the types of biologics (IFX or ADA) administered between the 2 groups. The CD onset age was 20 ± 7 years in the LOR group and 26 ± 10 years in the non-LOR group (*P* = .02), and the respective ages at the time of introducing biologics were 26 ± 9 and 36 ± 12 years (*P* = .006). Although the CRP levels at 1 year after the introduction of biologics did not differ significantly between the 2 groups, those at the time of introducing biologics were 28.0 ± 21.5 mg/L in the LOR group and 13.3 ± 15.3 mg/L in the non-LOR group, showing a significant difference (*P* = .002) (Table 3). As the correlation coefficient between age at CD onset and age at the time of introducing biologics was high at 0.734, multivariate analysis was performed with onset age as an explanatory variable. CRP levels at the time of introducing biologics (odds ratio [OR], 1.5; 95% confidence interval [CI], 1.04–2.06; *P* = .02) and age at CD onset (OR, 1.1; 95% CI, 1.01–1.20; *P* = .03) were identified as predictive factors for LOR.

**Table 3:**
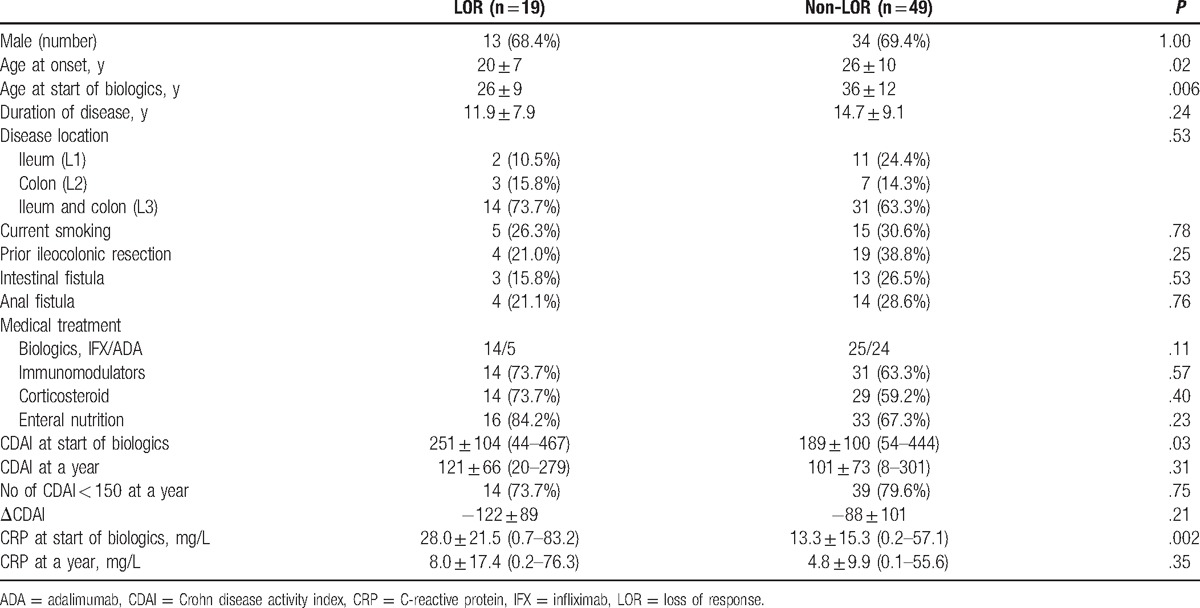
Potential risk factors for loss of response to biologics.

## 4 Discussion

In the present study, patients who achieved clinical remission at 1 year accounted for 69% of the IFX group and 90% of the ADA group. No significant difference was observed, and the therapeutic effects at 1 year were similar in the IFX and ADA groups. In a retrospective study comparing the efficacies of IFX and ADA in patients naive to biologics, although no significant difference was observed in numbers of patients undergoing surgery during the 18-month follow-up period after introduction, surgery tended to be less frequently performed in the IFX group.^[5]^ Successful mucosal healing is an important condition for maintaining long-term remission. A study of 54 CD patients receiving IFX showed that, although the presence or absence of mucosal healing in the large intestine did not contribute to the risk of requiring surgery, successful mucosal healing in the ileum was associated with a reduced risk and was thus important for improving the long-term prognosis.^[6]^ Combination therapy with IFX and immunomodulators also reportedly produces a higher remission rate than monotherapy with IFX and, furthermore, is more likely to be effective in the early stage after CD onset.^[7]^ The present study identified no difference in clinical remission status at 1 year between patients with and without the concomitant use of immunomodulators (data not shown).

Our study included only patients who continuously received the same biologic agent for 1 year or longer, and we assessed LOR both at and beyond 1 year after the introduction of biologics. There were no significant differences in event-free rates or secondary LOR between the 2 groups. Secondary LOR to IFX for CD reportedly occurs in 50% of cases.^[8,9]^ Regarding LOR to ADA, Billioud et al^[10]^ reported that the annual risk of experiencing secondary LOR to ADA was 20.3% per patient-year. In patients with secondary LOR to ADA, administration of this agent at a dose increased to 80 mg every 2 weeks or at a dose of 40 mg weekly was shown to be effective. However, tertiary LOR occurred in 56.8% of patients.^[11]^ Moreover, in a multicenter retrospective study comparing incidence rates of secondary LOR among 3 groups, that is, antitumor necrosis factor (TNF) therapy-naive patients treated with IFX, anti-TNF therapy-naive patients treated with ADA, and patients with prior anti-TNF exposure who were treated with ADA, although there was no significant difference between the anti-TNF therapy-naive patients treated with IFX and ADA, the incidence rates of secondary LOR were significantly higher in the patients with prior anti-TNF exposure administered ADA. Furthermore, the median time to secondary LOR was significantly longer in the anti-TNF therapy-naive patients treated with IFX than in either of the other 2 groups.^[12]^ Hibi et al reported that one of the causes of LOR is decreased IFX concentrations. The factors reported to reduce IFX concentrations include the production of antibodies to IFX, blood TNF-α concentrations before IFX therapy, and genetic polymorphism of Fcγ receptors.^[4]^

Hibi et al described an association between LOR and CRP. The blood trough concentrations of IFX were 1 μg/mL or higher in 80% of patients with a CRP level below 5 mg/L, whereas the concentrations decreased to less than 1 μg/mL in 60% to 80% of patients with a CRP level above 5 mg/L. Thus, a CRP level exceeding 5 mg/L allows the prediction of a subsequent decrease in blood trough concentrations of IFX to less than 1 μg/mL and can predict LOR.^[4]^ In the present study as well, CRP levels at the time of introducing biologics were higher in the LOR than in the non-LOR group and, furthermore, were identified as an independent risk factor for LOR. The cumulative secondary LOR rate was significantly higher in patients with a CRP level above 5 mg/L at the time of introducing biologics, and we consider CRP to be an important predictor of LOR. Although criteria and requirements for withdrawal of biologics are currently controversial, CRP may be an important factor in assessing the advisability of withdrawal of biologics. In CD patients who have received IFX for at least 1 year, a prospective, randomized, double-blind, placebo-controlled study (the STOP IT study) is currently underway to assess remission maintenance rates at 48 weeks after IFX is withdrawn, when the subjects have achieved a CDAI of less than 150, negativity for CRP, and endoscopic remission. The results of this study are eagerly awaited.^[13]^ A CRP level of more than 5 mg/L is also reportedly a factor independently predicting relapse after withdrawal of biologic agents.^[14,15]^

The present study has limitations. First, it is a single-center retrospective cohort study. Second, the sample size is small. Third, the follow-up periods varied, though not significantly, between the IFX and ADA groups, tending to be longer in the former. As described above, this is attributed to the 8-year difference in the time when IFX and ADA were listed on the National Health Insurance Price List in Japan. To avoid this bias, we limited observation period to maximum 6 years.

## 5 Conclusion

The efficacies of IFX and ADA were similar at 1 year after starting administration, and neither the event-free rate nor the secondary LOR rate during the 5-year period after introduction of biologics differed significantly between the 2 groups. Even among those who had achieved clinical remission at 1 year after the introduction of biologics, there were patients who experienced LOR in subsequent years. CRP was confirmed to be an important predictive factor for LOR. Thus, we consider not only low CDAI but also negativity for CRP to be essential for clinical remission.
